# Supplementary material for: An immortal porcine preadipocyte cell strain for efficient production of cell-cultured fat
Source: Commun Biol. 2023 Nov 25;6:1202. doi: 10.1038/s42003-023-05583-7 (PMC10676435; doi:10.1038/s42003-023-05583-7)
Supplement: Supplementary file 2 — Supplementary information [file 42003_2023_5583_MOESM2_ESM.pdf]

An immortal porcine preadipocyte cell strain for efficient production of cell-cultured fat

YUN-MOU Cheng[1,2,3], PENG-CHENG Hong[1,2,3], MING-MEI Song[1,2], HAI-NING Zhu[1,2], JING Qin[1,2], ZENG-DI Zhang[1,2], HAO Chen[1,2], XING-ZHOU Ma[1,2], MENG-YUAN Tian[1,2], WEI-YUN Zhu[1,2], ZAN Huang[1,2,*]

[1]Laboratory of Gastrointestinal Microbiology, Jiangsu Key Laboratory of Gastrointestinal Nutrition and Animal Health, College of Animal Science and Technology, Nanjing Agricultural University, Nanjing, China

[2]National Center for International Research on Animal Gut Nutrition, Nanjing Agricultural University, Nanjing, China

[3]These authors contributed equally
*Correspondence: Z.H. (huangzan@njau.edu.cn)

Supplementary Figures 1-9
Supplementary Table 1

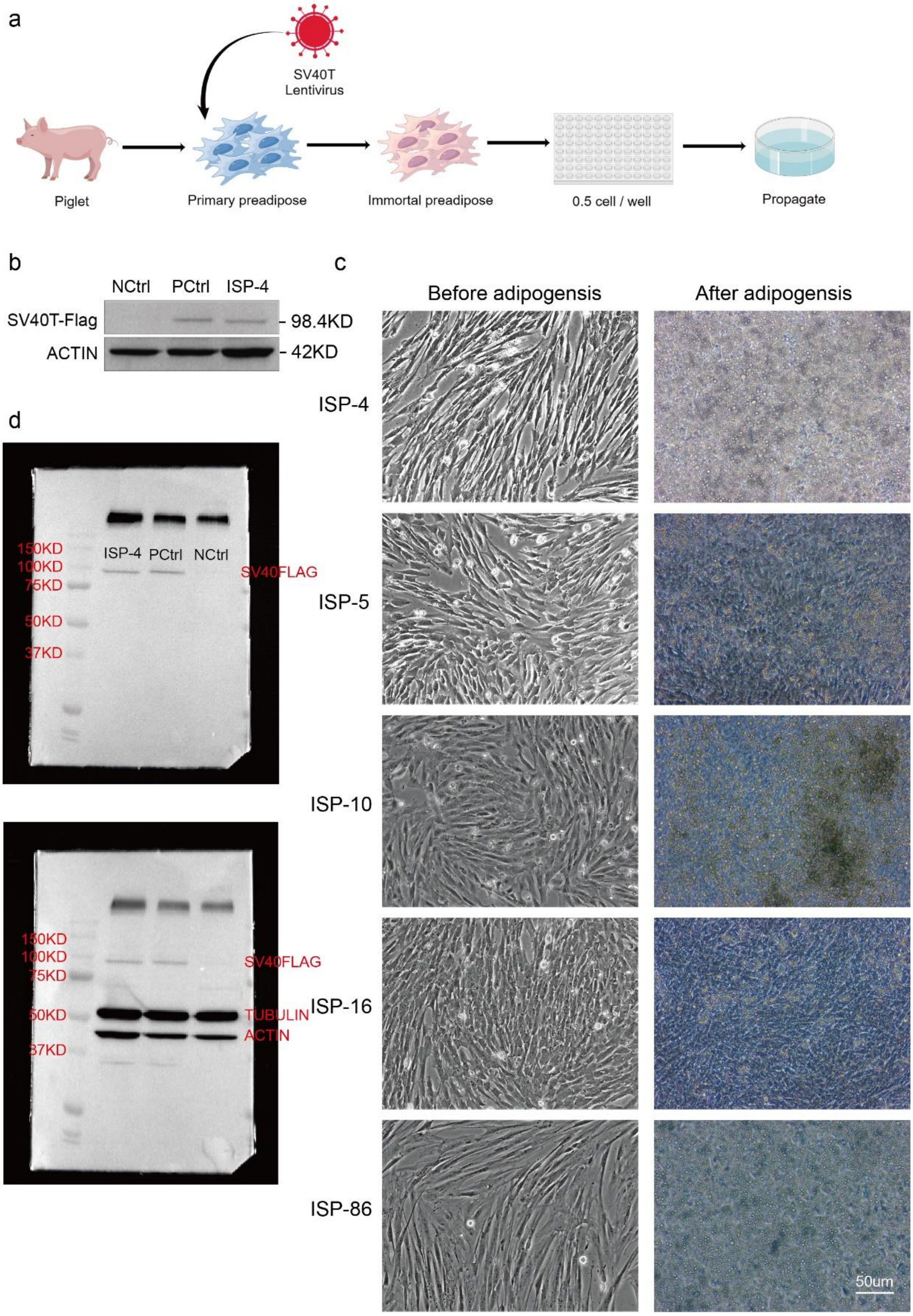

Supplementary Figure.1 Establishment of the immortalized swine preadipocytes cell strain.

a. Schematic representation of the establishment of swine pre-adipocyte cell strains (Created by Figdraw).
b. Immunoblot of SV40T protein expression in the ISP-4 cell strain.

c. Representative brightfield microscope images of isolated cell strains with adipogenic potential. Cell stains were adipogenic differentiated with 4+4 method. Scale bar = 50μm.
d. Uncropped immunoblotting in b.

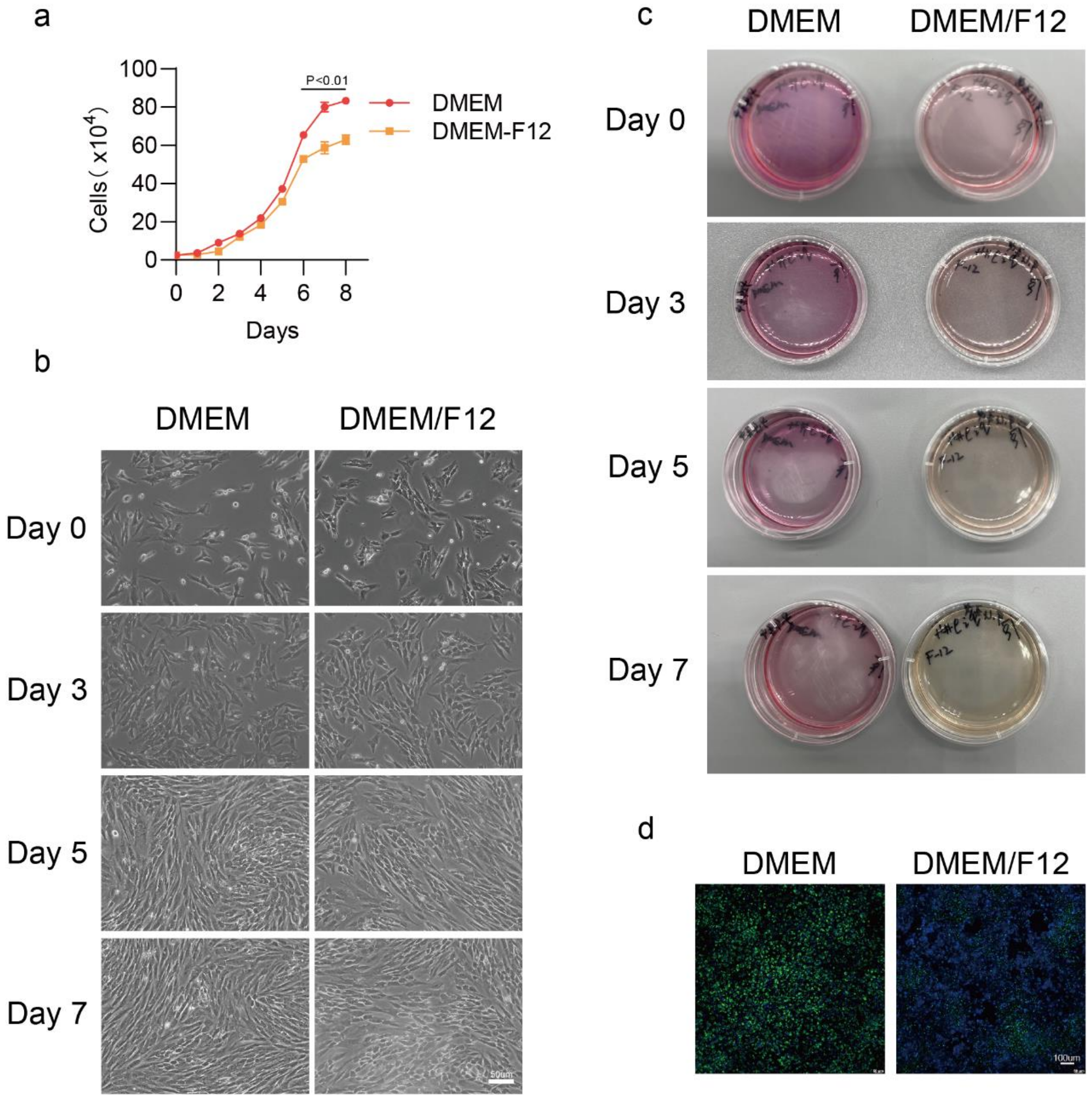

Supplementary Figure.2 Optimization of Culture Medium for ISP-4 Cell Line.

10^4 ISP-4 cells were seeded in 35mm dishes and cultured in either DMEM or DMEM/F12 medium supplemented with 10% FBS, with medium changes every 2 days. The cells were photographed and counted on the indicated days, and adipogenic differentiation was performed with the 4+4 protocol on day 8.

a. The growth curve of ISP-4 cells cultured in DMEM or DMEM/F12 medium (n=3 biologically independent samples).

b. Representative bright field images of ISP-4 cells on the indicated days are shown. Scale bar = 50 μm.

c. The DMEM/F12 medium was more acidic compared to DMEM medium when culturing ISP-4, indicated by the color of phenol red of medium.

d. Representative fluorescent images of ISP-4 cells differentiated with DMEM or DMEM/F12 medium are shown, with BODIPY (green) and Hoechst (blue) staining. Scale bar = 100 μm.

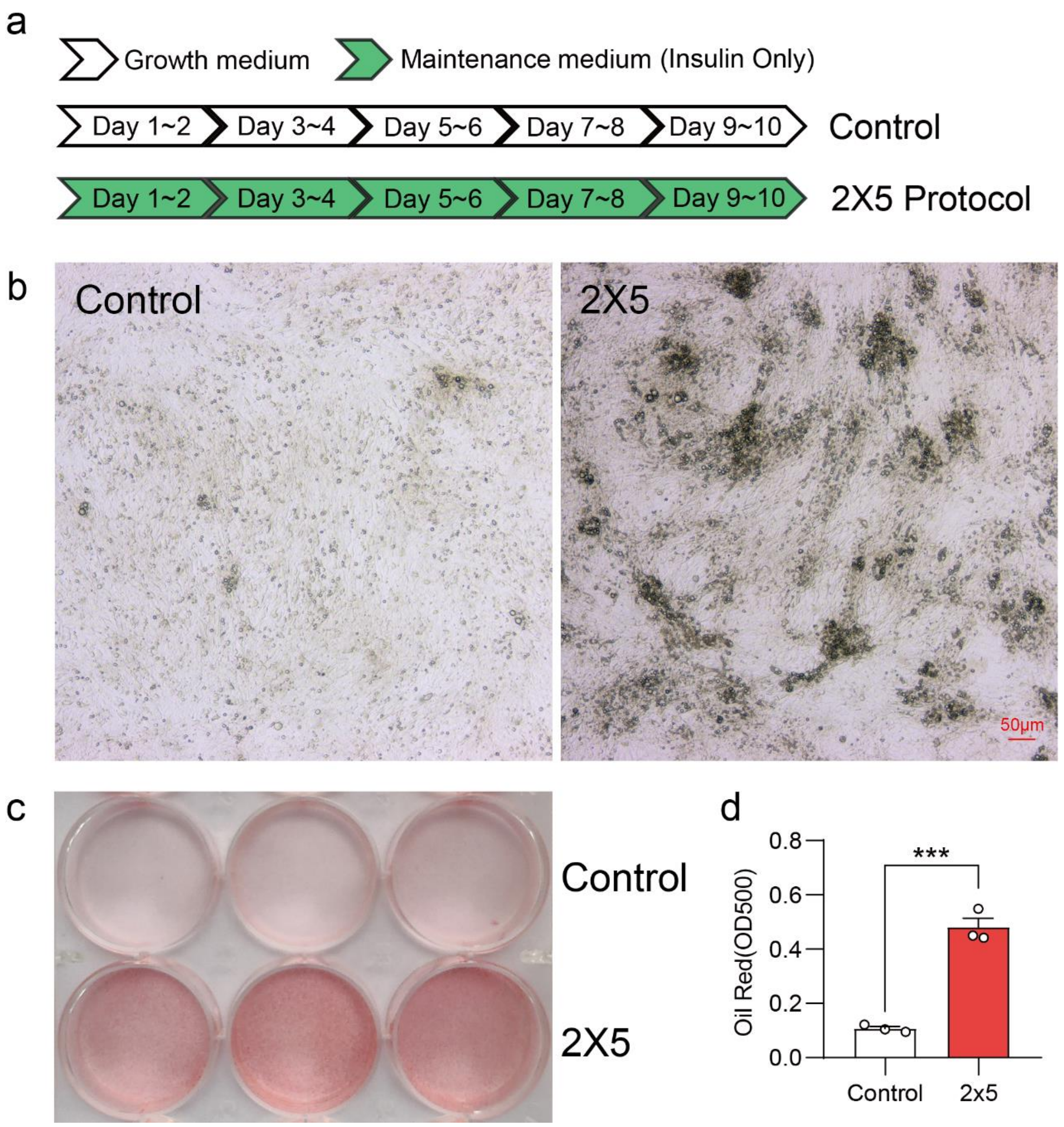

Supplementary Figure.3 ISP-4 cannot differentiate without stimulation.

a. Overview of workflow for adipogenic differentiation methods of ISP-4. Cells were cultured in DMEM+10%FBS with or without Insulin for 10 days, medium were changed every 2 days.

b. Representative bright field images of ISP-4 cells after 10 days of culture with indicated methods. Images were taken with a 4x objective lens on a bright-field microscope. Scale bar = 50 μm.

c. Image of Oil-Red stained ISP-4 cells, which differentiated as described in a.

d. Oil-Red was extracted with isopropanol and measured at 500nm (n=3 biologically independent samples).

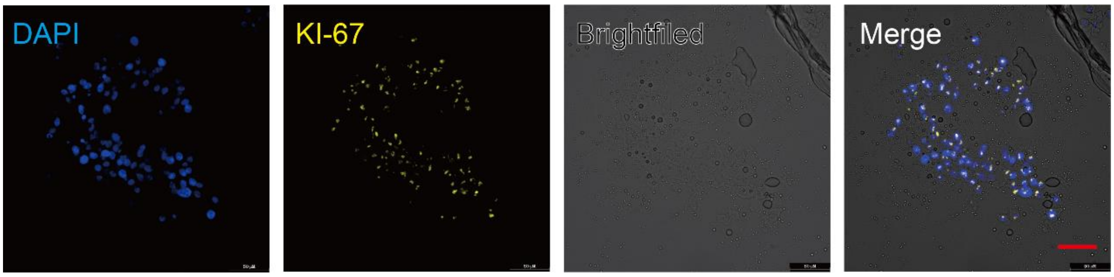

Supplementary Figure.4 ISP-4 still proliferate in alginate hydrogel

Immunofluorescent staining of day0 ISP-4 alginate hydrogel. Yellow = KI-67(Abclonal, Cat#A2094, Lot#5500008522), blue = DAPI, Scale bar = 50μm.

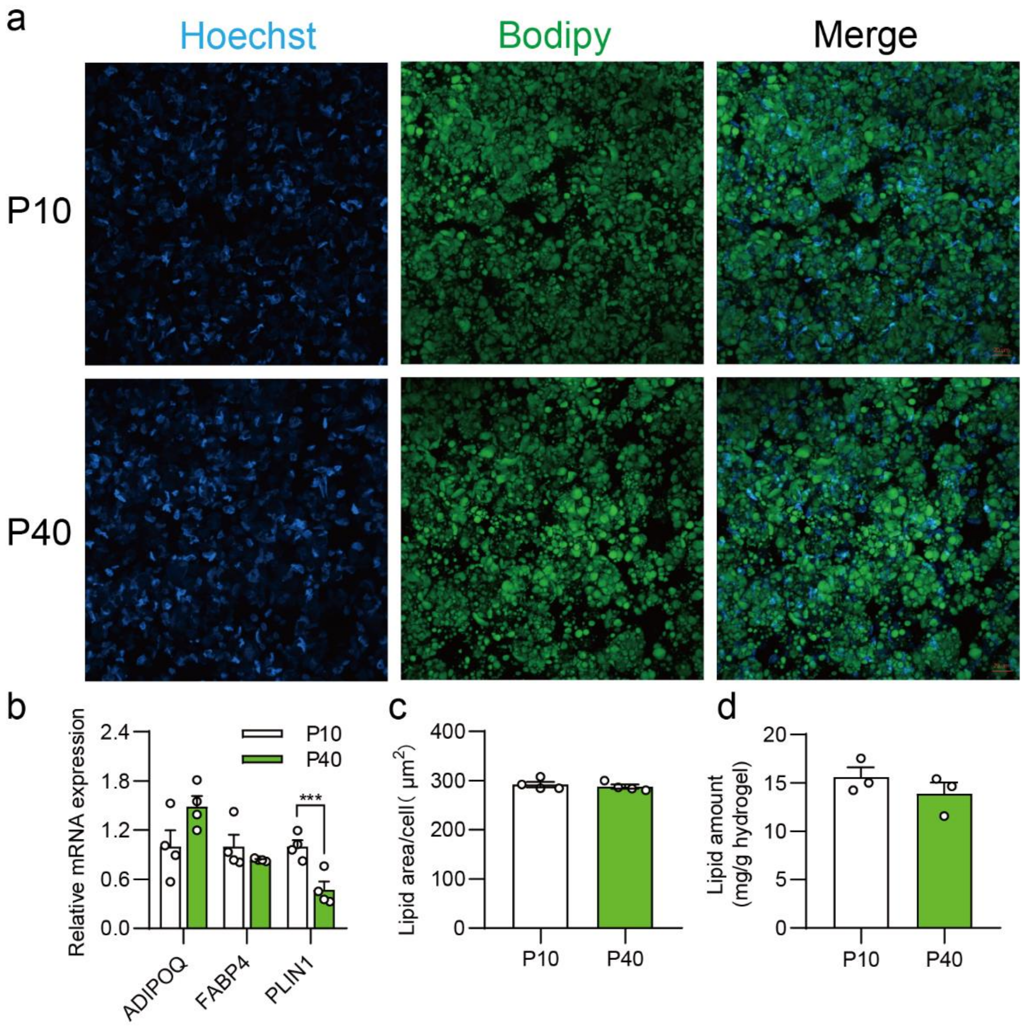

Supplementary Figure.5 Related to Figure.3

a. Representative images of adipogenic differentiated P10 and P40 ISP-4 in alginate hydrogel with a “4+4” protocol. Images were captured using confocal microscopy with maximum intensity projection. Scale bar = 20 μm.

b. Expression levels of adipose-specific genes in alginate hydrogels with P10 and P40 ISP-4. Data were measured with RT-qPCR (n=4 biologically independent samples).

c. The lipid content was quantified by measuring the BODIPY and Hoechst fluorescence area in figures A (n=4 biologically independent samples).

d. The lipid content in ISP-4 alginate hydrogel was measured by triglyceride content assay kit (n=3 biologically independent samples).

All numerical values are presented as mean ± SEM. Two-tailed unpaired Student’s t-test *p*-values are indicated as ***P ≤ 0.001.

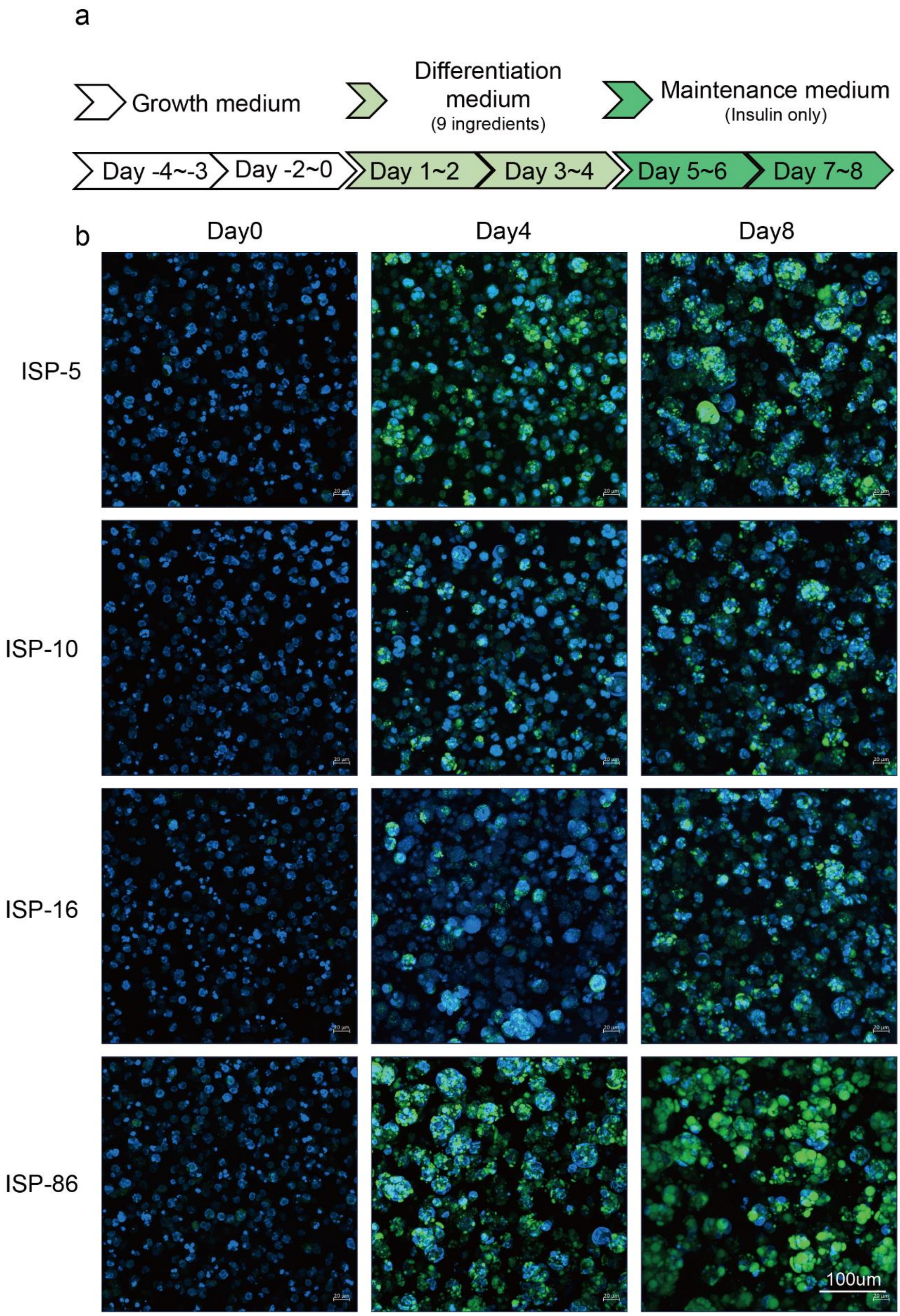

Supplementary Figure.6 Adipogenic differentiation of other porcine preadipocyte cell strains in alginic hydrogels.

a. Schematic illustration of the adipogenic differentiation process for alginic hydrogels with various cell strains.

b. Representative fluorescence micrographs showing the adipogenic differentiation of the indicated cell strains. BODIPY staining is shown in green, Hoechst staining in blue. Scale bar = 100 μm.

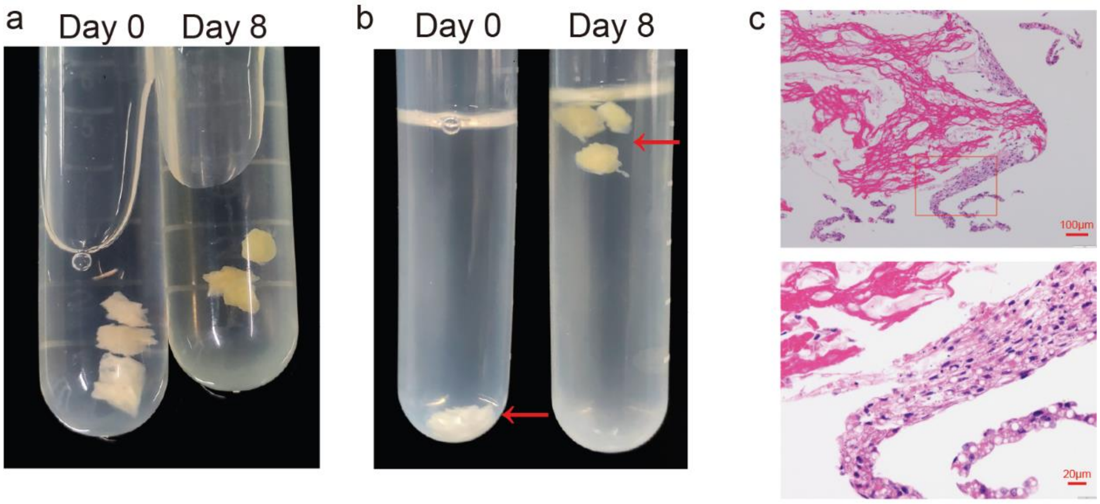

Supplementary Figure.7 ISP-4 can adipogenic differentiated on the peanut wire-drawing protein scaffold.

a-b. The color(A) and density(B) changed before and after adipogenic differentiation of ISP-4 on peanut wire-drawing protein scaffold.

c. Representative H&E staining of differentiated ISP-4 on peanut wire-drawing protein scaffold at 4x(top) and 10x(bottom) magnification.

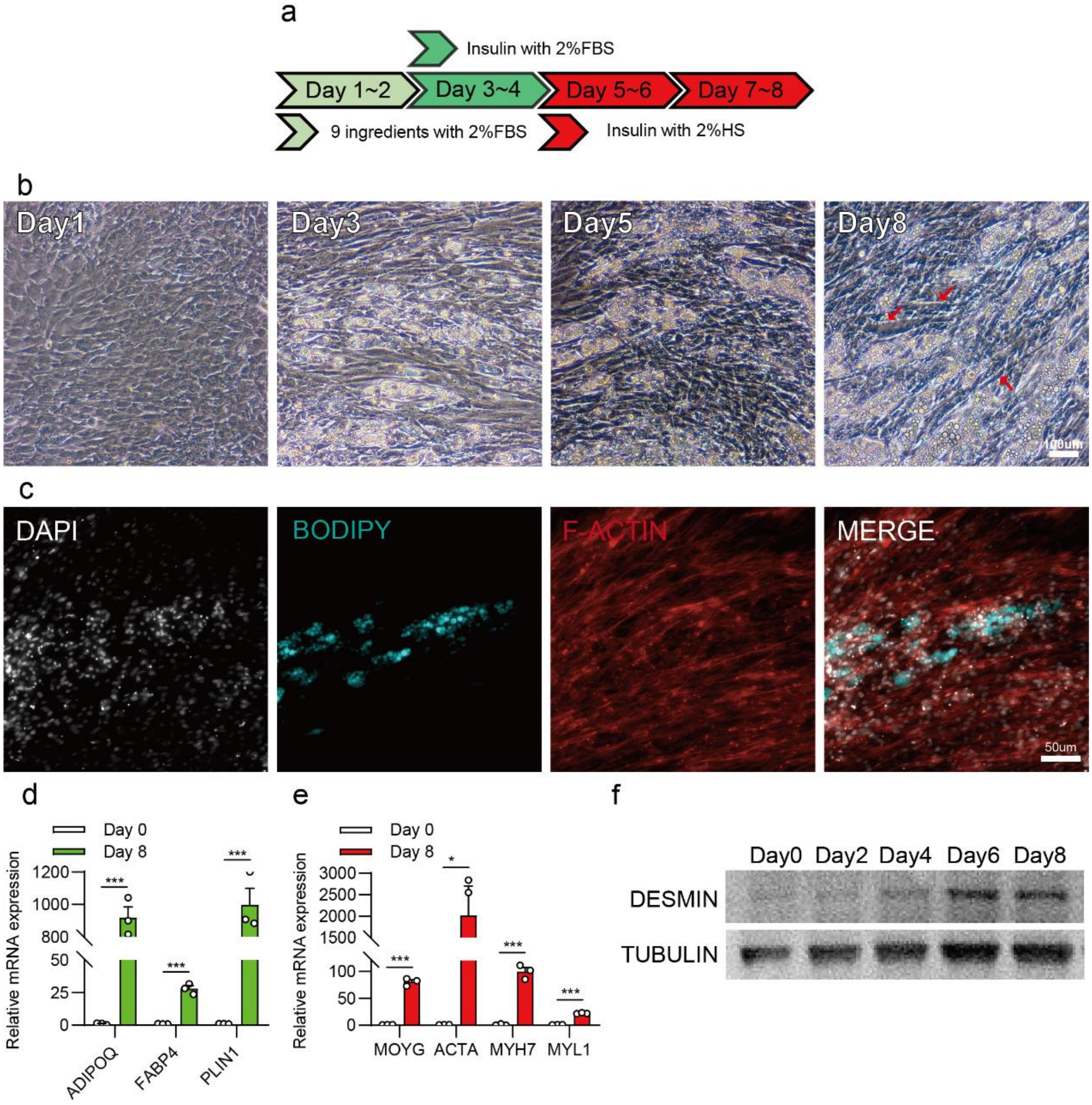

Supplementary Figure.8 ISP-4 can co-differentiated with porcine myoblast cells.

A. Workflow overview: ISP-4 was co-cultured with PMSC cells, cells were adipogenic differentiated with ADM for 2 days, then AMM for other 2 days. Subsequently, myogenic differentiation was induced in DMEM with 2% HS for an additional 4 days. The medium was changed every 2 days.

B. Representative images of co-cultured ISP-4 and C2C12 cells at indicated time points after treatment. Notice the hypertrophy and elongation of the mature muscle cells. Scale bar = 100μm.

C. Representative fluorescence images of co-differentiated ISP-4 and PMSC cells. Nuclei were stained with DAPI, lipid with BODIPY, and F-ACTIN with phalloidin. Scale bar = 50μm.

D-E. Expression of adipose-specific genes (D) and muscle-specific genes (E) for the samples presented in Figure B. The data were measured by RT-qPCR and normalized against day 0 (n=3 biologically independent samples).

F. Immunoblotting against DESMIN at indicated time point of co-differentiation, TUBULIN was used as loading control.

All numerical values are expressed as mean ± SEM. Two-tailed unpaired Student's t-test

p-values are indicated as $*P \leqslant 0.05$, $***P \leq 0.001$.

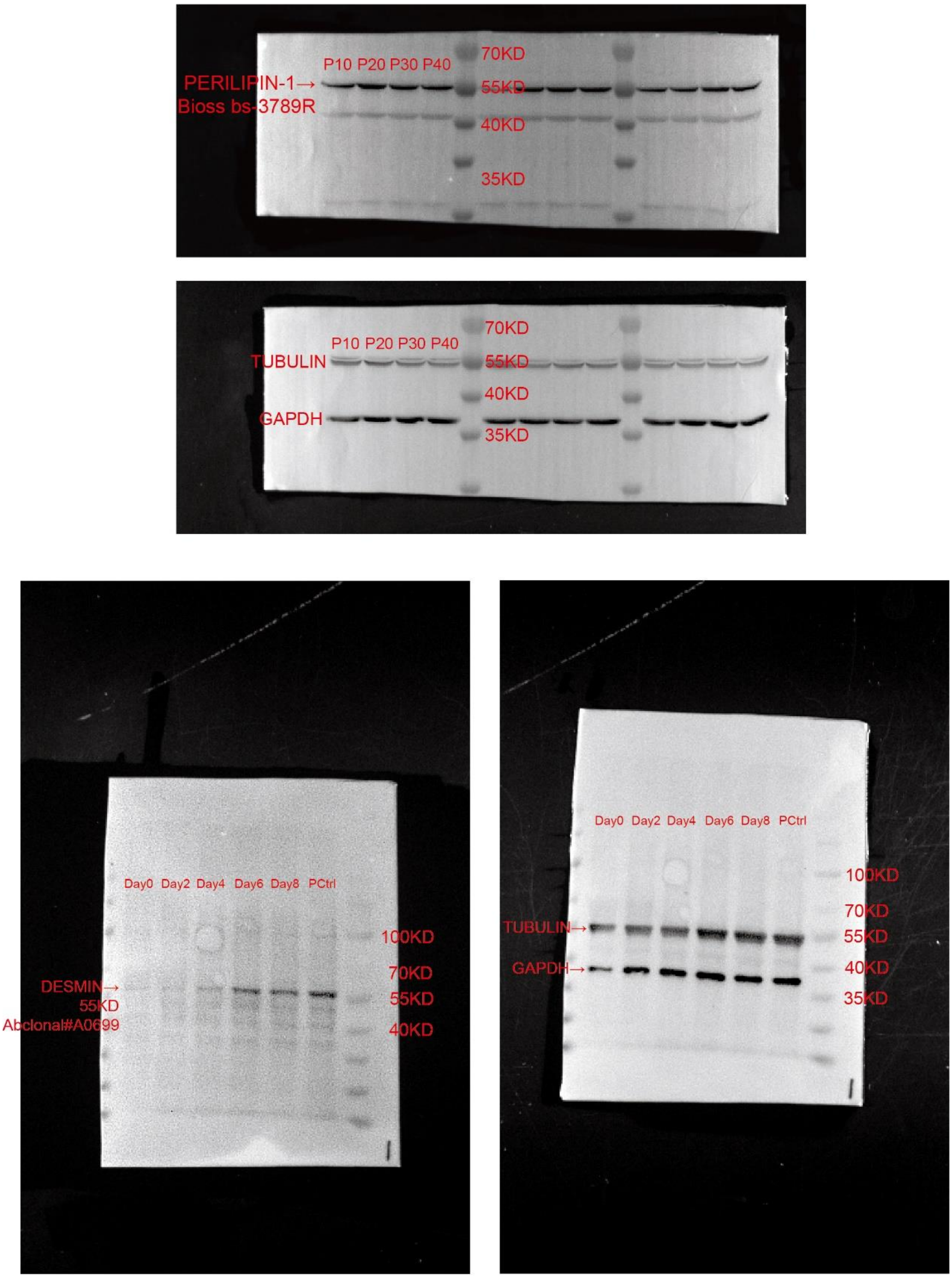

Supplementary Figure.9 Uncropped immunoblotting.

Primer	Sequence（5’ to 3’）
36B4-F	AGATGCAGCAGATCCGCAT
36B4-R	GTTCTTGCCCATCAGCACC
Mice Myh1-F	GCCCAGTGGAGGACAAAATA
Mice Myh1-R	TCTACGTGCTCCTCAGCAT
Mice Myod1-F	AACTGCTCTGATGGCATGATGGATT
Mice Myod1-R	CTCGACACAGCCGCACTCTT
Mice Myog-F	GTCCCAACCCAGGAGATCATT
Mice Myog-R	AGTTGGGCATGGTTTCGTCT
Pig FABP4-R	CCCTGGTACAGGTGCAGAAG
Pig FABP4-F	TGGTAGCCGTGACACCTTTC
Pig PLIN1-F	GCTTACGAGAAGGGCATCCA
Pig PLIN1-R	TGGAGGGCCGGTATCTTTTC
Pig ADIPOQ-F	CGAGAAGCCTGGAGCACTAC
Pig ADIPOQ-R	CTTTTCTGCCCGGGATTCCT
Pig MYOG-F	CGAGACCTCCGCTACCGA
Pig MYOG-R	AGGGTCAGCTGTGAGCAGAT
Pig ACTA-F	GCCGGAGAGCAGCAGAAA
Pig ACTA-R	GACCCATACCCACCATGACG
Pig MYH7-F	GTTTGCCAACTATGCTGGGG
Pig MYH7-R	TGGAGCGCAAGTTGGTCATC
Pig MYL1-F	GGCACAAATCCCACCAATGC
Pig MYL1-R	AAGACACGCAGACCCTCAAC

Supplementary Table 1 Primers for RT-qPCR
