# Supplementary material for: An immortal porcine preadipocyte cell strain for efficient production of cell-cultured fat
Source: Commun Biol. 2023 Nov 25;6:1202. doi: 10.1038/s42003-023-05583-7 (PMC10676435; doi:10.1038/s42003-023-05583-7)
Supplement: Supplementary file 3 — Description of additional supplementary files [file 42003_2023_5583_MOESM3_ESM.docx]

Description of Additional Supplementary Files

**File name:** Supplementary Data 1

**Description:** The source data behind the graphs in the paper
